# Effect of home blood pressure monitoring for blood pressure control in hypertensive patients taking multiple antihypertensive medications including fimasartan (the FORTE study)

**DOI:** 10.1186/s40885-020-00154-y

**Published:** 2020-12-15

**Authors:** Jung-Yeon Choi, Kwang-il Kim, Cheol-Ho Kim

**Affiliations:** 1 Department of Internal Medicine, Seoul National University Bundang Hospital, Seongnam, Republic of Korea; 2 Department of Internal Medicine, Seoul National University College of Medicine, Seoul, Republic of Korea

**Keywords:** Blood pressure, Hypertension, Korea

## Abstract

**Background:**

Although recent hypertension guidelines recommend home blood pressure (HBP) monitoring, its effect in clinical practice is not well known. This study aimed to identify current HBP measurement status and obstacles and their efficacy on blood pressure (BP) control.

**Methods:**

Sixty-three intervention and 61 control centers with 2483 (mean age: 58.0 years, 56.0% male) drug-naïve stage 2 hypertensive patients or patients requiring second anti-hypertensive medications were included. The intervention group was instructed to measure HBP twice a day for 7 days from the scheduled visit at 4, 8, and 12 weeks.

**Results:**

At the end of 12 weeks, 842 (68.7%) and 807 (64.15%) patients of the control and intervention groups, respectively, achieved a target BP. The odds ratio (OR) for improving BP control of HBP was 0.836 (95% confidence interval [CI]: 0.694–1.007). Among intervention group, clinic BP of the subgroup those measured their HBP at least once well controlled compared to subgroup those not measured their HBP at all (OR 1.602, 95% CI: 1.182–2.172). Only 19.17% (*n* = 476) had a home sphygmomanometer, and among those, 26.89% measured their BP at least once a week and 34.87% did not measure the BP at all. The obstacles of HBP measurement were lack of awareness of its importance (40.83%), lack of confidence on how to measure BP and maintain the measurement (37.04%), and difficulty in selecting an appropriate device (14.41%).

**Conclusions:**

HBP measurement alone did not improve BP control, but better compliance with the HBP measurement resulted in improved BP control.

**Trial registration:**

ClinicalTrials, NCT03254914, Registered 21 August 2017.

## Background

Hypertension is a major risk factor for stroke and premature cardiovascular diseases [1]. For diagnostic and prognostic evaluation, accurate blood pressure (BP) measurement is important. Given that BP significantly varies depending on the measurement protocol or place, it is recommended that BP be measured using a standard method. BP has traditionally been measured in the clinical setting (clinic BP [CBP]) with the auscultatory method and a mercury sphygmomanometer since it is regarded as the standard instrument. Clinicians became aware of problems of white-coat syndrome and masked hypertension and started referencing not only CBP but also home blood pressure (HBP) or 24-h ambulatory blood pressure (24-h ABP). Previous studies reported that HBP or 24-h ABP is more useful in predicting future stroke or cardiovascular events. Thus, HBP measurement became widely used as it is easy to use, inexpensive, useful in predicting future stroke or cardiovascular events, and accurate when an automated sphygmomanometer is used [2–4]. Moreover, the act of measuring one’s HBP is known to motivate patients to have more interest in managing their hypertension and to positively affect BP control rates by improving compliance with BP-lowering medications [5]. Notwithstanding, it is only recently that HBP has been actively used and accepted as a reference for the management of hypertension in an actual clinical setting [6].

The Korean guidelines for hypertension treatment have also stated the importance of HBP [7]. However, according to the latest survey results, it is still not actively recommended that physicians refer to HBP [8]. Obstacles to the universal use of HBP include the absence of effective educational materials for patients, concerns regarding accuracy, patients’ prejudice toward HBP measurement, and a lack of patients’ confidence, and there is insufficient study data on hypertensive Korean patients [8, 9].

In other countries, the use of HBP is increasing, but it is also recommended to a limited number of patients suspected of having hypertension induced by white-coat syndrome or masked hypertension, or to be used as an alternative when 24-h ABP cannot be measured [10–14]. Meanwhile, the Japanese guideline has actively adopted the use of HBP especially if there is a disparity between HBP and CBP, and large-scale clinical epidemiological studies have been carried out [15–17].

Hypertension is the leading modifiable risk factor for cardiovascular disease and premature death worldwide, accounting for > 20% of all deaths in older adults aged ≥70 years, and its prevalence is increasing [18, 19]. The importance of BP control can be further emphasized in Korea, since the proportion of older adults has doubled in only 17 years from 7% (an aging society) in 2000 to 14% (an aged society) in 2017, and it is expected to grow up to 20% (a post-aged society) by 2026 [20]. Particularly, if telemedicine is put in place, it will become mandatory to measure HBP to manage hypertension in patients living in remote or rural areas with inadequate medical infrastructure [21]. However, little is known about the efficacy of HBP measurement and real-world HBP measurement in Korea.

Therefore, this study aimed to compare BP control rates between the intervention (HBP measured) and control (HBP unmeasured) groups at 12 weeks in order to identify the effectiveness of HBP measurement for BP control, and to analyze the BP control rate according to actual HBP measurement compliance. We also performed a survey to identify the current status and obstacles of HBP measurement in Korea and to evaluate the safety and effectiveness of fimasartan.”

## Methods

### Study sites and patients’ inclusion criteria

This work was a multi-center, cluster-randomized, prospective observational study. Randomization was implemented at cluster (study center) level and stratified was performed by the type of center (clinic or hospital). The inclusion criteria were as follows: 1) medical institutions located in the Republic of Korea, 2) sites with facilities (separate consulting rooms, chairs, or beds) where CBP is measured according to the clinical practice guidelines under the supervision of nurses or medical staff, and 3) sites with personnel who can educate participants on how to measure HBP and can contact via text message or calls. The randomization list was produced by a designated statistician using SAS software version 9.2 (SAS Institute, Cary, NC, USA), and information about randomization was opened at cluster level only.

Staff at the study centers asked their patients to voluntarily participate in this study, allocated numbers to those who signed the informed consent, and checked the inclusion and exclusion criteria. The inclusion criteria were as follows: 1) age > 19 years, 2) signed informed consent, and 3) use of at least ≥2 antihypertensive medications including fimasartan. Detailed inclusion and exclusion criteria are presented in the Additional file 1. This study protocol was reviewed and approved by the institutional review board (IRB) of Seoul National University Bundang Hospital (IRB number: B-1707/408–304).

From May 12, 2017 to August 162,017, 132 centers were initially planned to be enrolled in this study; of these, 6 centers withdrew their consent to participate, resulting in 126 remaining centers (64 intervention and 62 control sites). One center in both the intervention and control groups was excluded, because they did not enroll any participants. Finally, from August 31, 2017 to August 8, 2018, 1767 and 1787 participants from 63 intervention and 61 control sites were enrolled, respectively.

### CBP and HBP measurements

The same sphygmomanometers were used, and CBP was measured twice at 2-min intervals in a quiet, separated room after a 5-min rest according to the standard guideline [22]. The arm with the higher mean systolic pressure after measuring BP in both arms twice at the baseline point was used as the reference arm (if systolic pressures were the same in both arms, the arm with the higher mean diastolic pressure was used); and in subsequent visits, BP and pulse rates measured twice using the reference arm were collected.

In the intervention group, on Visit 1, patients were educated on how to measure HBP and provided educational materials, sphygmomanometer, manual, and BP diary. After Visit 1, participants were reminded through text message or calls (only if text message is unavailable) 8–10 days prior to the study visits. Participants were instructed to measure BP twice a day (twice an hour after waking up and twice before sleeping at 2-min intervals; total of four times) for 7 days from the scheduled visit and to record the BP readings along with pulse rates in the BP diary provided. Detailed measurement time and method are given in the Additional file 2.

Among the intervention group, to verify the effect of the compliance with the HBP measurement on BP control rate, those who never measured HBP and those who measured HBP at least once were classified subgroups. At enrollment, all participants were surveyed for existence of home sphygmomanometer, experience on HBP measurement, willingness to purchase a sphygmomanometer, and reason for non-purchase to identify the current status of HBP measurement in Korea.

### Outcomes

For the primary outcome, achievement of the target BP at 12 weeks was analyzed between the intervention and control groups. For the secondary outcome, achievement of the target BP at 12 weeks in the between the subgroups according to compliance was analyzed to determine whether the compliance with HBP affects the control of hypertension. Additionally, the current status of HBP in Korea was investigated with a structured survey.

For the safety analysis, we collected data for the incidence and characteristics of antihypertensive agent-associated adverse drug reactions (ADRs), serious adverse events, and serious adverse drug reactions (SADRs) and abnormal laboratory findings. Recruited patients were followed up three times at 4-week intervals for 12 weeks, and safety data were collected every time they visited the site. Detailed data collection schedule and items are presented in Additional file 3.

### Target BP and treatment

According to the Korean guidelines issued to primary healthcare institutions, basic target BP was < 140/90 mmHg based on CBP, but different standards were applied in people aged ≥80 years (< 150/90 mmHg), diabetic patients (< 140/85 mmHg), and patients with chronic kidney disease as indicated by proteinuria (urine albumin level ≥ 30 mg/d or ≥ 30 mg/g [albuminuria], urine protein level ≥ 150 mg/d or ≥ 150 mg/g [proteinuria]) (< 130/80 mmHg) [23].

Fimasartan administration was determined based on clinical need, and patients requiring ≥2 antihypertensive agents, including fimasartan, were enrolled. The choice of other antihypertensive agents and their doses were adjusted to reach the target BP while monitoring participants’ BP in accordance with the guidelines for clinical practice. Use of other agents/treatments necessary to treat diseases other than hypertension was not restricted, and drugs/treatments judged clinically required were all permitted. However, the following drugs were prohibited according to the precautions for use of fimasartan: 1) renin inhibitor (aliskiren) in patients with hypertension or moderate-to-severe renal disorders and 2) angiotensin-converting enzyme inhibitor in patients with diabetic nephropathy.

### Statistical analysis

Efficacy analysis was performed on participants who met the inclusion/exclusion criteria and had BP data on the enrollment and any post-enrollment point. The last observation carried forward method was used to impute missing BP data. Safety analysis was performed on all participants who took fimasartan at least once and provided safety follow-up information. Descriptive statistics are provided throughout, including mean and standard deviation for continuous variables and frequency and percentages for categorical variables. The difference in achievement of target BP at 4, 8, and 12 weeks between the control and intervention groups were assessed using the Cochran-Mantel-Haenzel test, and the level of hospital (clinic/hospital) was considered as a stratification factor. To compare the continuous variables, the chi-square test and Fisher exact test were used after the normality test with the Shapiro-Wilk test. The Wilcoxon rank sum test, independent t-test, Mann-Whitney U test, or logistic regression were also used to identify the confounding factors that may impact BP control. Sex, age, target organ damage, baseline BP, family history, metabolic status, existence of a home sphygmomanometer at baseline, and other potential confounding factors were analyzed together as confounding variables to statistically analyze the intervention effect. All statistical analyses were performed using SAS software version 9.4 (SAS Institute, Cary, NC, USA), and *p* < 0.05 was considered significant with two-tailed analysis. All data from the participants were de-identified and analyzed anonymously.

## Results

### Patients’ characteristics

**Fig. 1:**
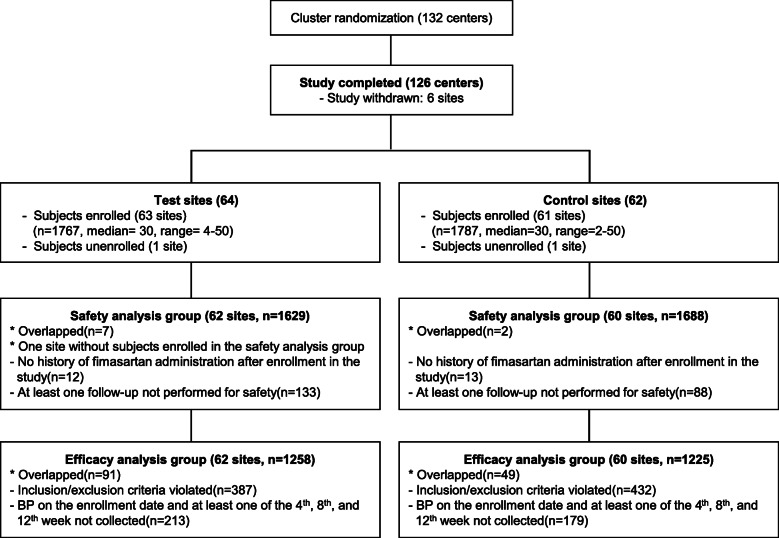
Flow of patients in the study

The percentages of participants who completed the 12-week follow-up were 83.6% (1478/1767) and 88.3% (1577/1787) for the intervention and control groups, respectively. The most common reason for withdrawal was lost to follow-up in both groups (53.31%). The numbers of participants included in the safety analysis group were 1629 (92.2%) and 1688 (94.5%) in the intervention and control groups, respectively. The numbers of patients included in the efficacy analysis group were 1258 (71.2%) and 1225 (68.6%) for the intervention and control groups, respectively (Fig. 1).

Patients’ baseline characteristics, including age, sex, and body mass index, were statistically different between the intervention and control groups. Additionally, for the confounding factors that could influence the severity of patients at baseline, the cardiovascular risk factor was more prevalent in the control group than in the intervention group, and target organ damage was more prevalent in the intervention group than in the control group. The control group tended to use more combinations of antihypertensive medications than the intervention group (Table 1).

**Table 1:** Comparison of the Baseline Demographic and Confounding Characteristics Between the Groups

	Control (***n*** = 1225)	Intervention (***n*** = 1258)	***P*** Values
Age (year)	58.85 (12.82)	57.26 (12.43)	***0.001*** ^W^
Sex (male/female)	724/501	666/592	***0.002*** ^C^
Body mass index (kg/m^2^)	25.39 (3.6)	25.74 (3.7)	***0.037***^***W***^
Baseline SBP (mmHg)	161.2 (14.57)	159.1 (15.16)	***< 0.001*** ^W^
Baseline DBP (mmHg)	97.4 (11.4)	97.7 (11.8)	0.345 ^W^
Antihypertensive medication (single/combination)	94/1131	203/1055	***< 0.001*** ^C^
Target organ damage^a^	122 (10.0%)	228 (18.7%)	***< 0.001*** ^C^
Cardiovascular risk factor	1120 (91.4%)	1112 (88.4%)	***0.012***^***C***^
Age (men ≥55 years, women ≥65 years)	641 (52.3%)	566 (45.0%)	***< 0.001*** ^C^
Male sex	724 (59.1%)	666 (52.9%)	***< 0.001*** ^C^
Smoking	249 (20.3%)	252 (20.0%)	0.855^C^
Dyslipidemia^b^	150 (12.2%)	220 (17.5%)	***< 0.001*** ^C^
FBS ≥100 mg/dL	149 (12.2%)	201 (16.0%)	***0.006***^***C***^
BMI ≥25 kg/m^2^	574 (50.0%)	693 (55.1%)	0.133^C^
Central obesity (men ≥90 cm, women ≥80 cm)	256 (20.9%)	326 (25.9%)	***0.003***^***C***^
Family history (men < 55 years, women < 65 years)	60 (4.9%)	29 (2.3%)	***< 0.001*** ^C^

Data are presented as mean (standard deviation) or number (%)

*SBP* systolic blood pressure, *DBP* diastolic blood pressure, *FBS* fasting blood glucose, *BMI* body mass index, *eGFR* estimated glomerular filtration rate, *LDL-C* low-density lipoprotein cholesterol, *HDL-C* high-density lipoprotein cholesterol

C: chi-square test

W: Wilcoxon rank sum test

^a^Target organ damage was defined as a composite of 1) stroke, transient ischemic attack, or vascular dementia; 2) left ventricular hypertrophy, angina, myocardial infarction, or heart failure; 3) albuminuria (albumin level > 30 mg/g) or chronic kidney disease (eGFR < 60 mL/min/1.73 m^2^); 4) peripheral vascular disease (ankle-brachial index < 0.9), pulse wave velocity > 10 m/sec, intimal thickness of the carotid artery > 1.0 mm, or large artery disease; and 5) stage 3 or 4 hypertensive retinopathy

^b^Dyslipidemia was defined as total cholesterol level ≥ 230 mg/dL, LDL-C level ≥ 150 mg/dL, HDL-C level < 40 mg/dL, or triglyceride level ≥ 200 mg/dL

### Effect of the HBP measurement

**Fig. 2:**
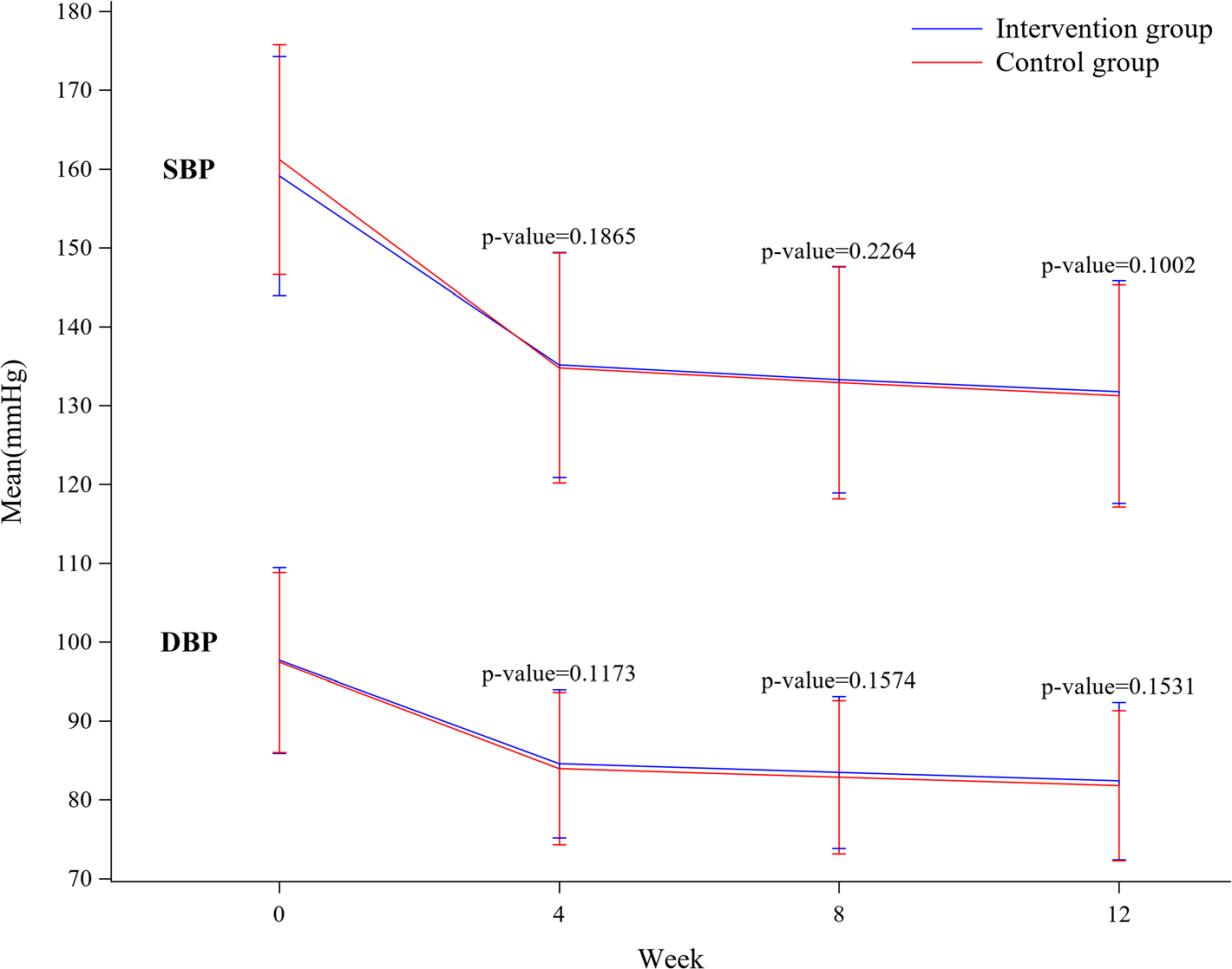
Changes in mean SBP and DBP by group at 4, 8, and 12 weeks. Each midpoint and rod represent the mean and standard deviation. Presented *p*-value is the result of the difference test between the groups using the mixed model for the repeated measured data. The SBP or DBP changes from baseline to each week are the response variables, and the group (intervention vs. control), baseline blood pressure level, level of hospital (clinic or hospital), time point (4/8/12 weeks), and interaction between the group and time point are the fixed effects. Moreover, the participants are the random effects. An unstructured covariance structure is assumed to model the within participant errors. SBP, systolic blood pressure; DBP, diastolic blood pressure

During the 12-week study period, 530 (55.91%), 686 (61.91%), and 842 (68.73%) patients in the control group and 534 (51.15%), 686 (57.74%), and 807 (64.15%) patients in the intervention group patients achieved the target BP at 4, 8, and 12 weeks, respectively (*p* = 0.0386, 0.0544, and 0.0164, respectively). Although there was a significant difference in the control rate of target BP between the groups, systolic and diastolic BP did not differ for 12 weeks (Fig. 2).

In order to determine the effectiveness of the introduction of and encouraging HBP measurement for BP control, we used multiple logistic regression models to determine the adjusted effect. After adjusting the potential cofounding factors, which could affect BP control and basic demographic characteristics, the results showed that the HBP measurement did not affect BP control (Table 2).

**Table 2:** Effect of Home Blood Pressure Monitoring on Blood Pressure Control

	Odds ratio		*P*-value
	Point Estimate	95% CI	
Age (years)	1.013	(1.005, 1.022)	0.0022
Sex	0.772	(0.632, 0.943)	0.0111
Target organ damage^a^	0.870	(0.668, 1.133)	0.3005
Home sphygmomanometers at baseline	0.959	(0.759, 1.212)	0.7262
Baseline SBP	0.990	(0.983, 0.997)	0.0066
Baseline DBP	0.991	(0.981, 1.001)	0.0845
Dyslipidemia^b^	0.968	(0.749, 1.252)	0.8046
FBS ≥100 mg/dL	0.715	(0.550, 0.929)	0.0121
Central obesity (men ≥90 cm, women ≥80 cm)	1.237	(0.993, 1.542)	0.0578
Family history of cardiovascular disease (men < 55 years, women < 65 years)	1.436	(0.875, 2.357)	0.1519
Intervention vs. control	0.836	(0.694, 1.007)	0.0586

*SBP* systolic blood pressure, *DBP* diastolic blood pressure, *FBS* fasting blood glucose, *BMI* body mass index, *eGFR* estimated glomerular filtration rate, *LDL-C* low-density lipoprotein cholesterol, *HDL-C* high-density lipoprotein cholesterol

^a^Target organ damage was defined as a composite of 1) stroke, transient ischemic attack, or vascular dementia; 2) left ventricular hypertrophy, angina, myocardial infarction, or heart failure; 3) albuminuria (albumin level > 30 mg/g) or chronic kidney disease (eGFR < 60 mL/min/1.73 m^2^); 4) peripheral vascular disease (ankle-brachial index < 0.9), pulse wave velocity > 10 m/sec, intimal thickness of the carotid artery > 1.0 mm, or large artery disease; and 5) stage 3 or 4 hypertensive retinopathy

^b^Dyslipidemia was defined as total cholesterol level ≥ 230 mg/dL, LDL-C level ≥ 150 mg/dL, HDL-C level < 40 mg/dL, or triglyceride ≥200 mg/dL

### Effect of compliance with the HBP measurement

Among 1258 patients in the intervention group who were introduced, educated, and encouraged to measure HBP, the effect of the compliance on BP control was analyzed. After adjusting for potential confounding factors and demographic variables, the BP of participants who actually measured HBP at least once tended to be well controlled statistically (Table 3).

**Table 3:** Compliance with Home Blood Pressure Monitoring and Blood Pressure Control

	Odds Ratio		
	Point Estimate	95% CI	*P*-value
Age	1.009	(0.997, 1.021)	0.1237
Sex	0.816	(0.616, 1.080)	0.1546
Target organ damage^a^	0.792	(0.568, 1.104)	0.1691
Home sphygmomanometers at baseline	0.900	(0.665, 1.217)	0.4923
Baseline SBP	0.998	(0.988, 1.008)	0.6615
Baseline DBP	0.986	(0.972, 1.000)	0.0519
Dyslipidemia^b^	0.804	(0.571, 1.131)	0.2105
FBS ≥100 mg/dL	0.601	(0.421, 0.857)	0.0050
Central obesity (men ≥90 cm, women ≥80 cm)	1.460	(1.070, 1.992)	0.0171
Family history of cardiovascular disease (men < 55 years, women < 65 years)	1.429	(0.584, 3.495)	0.4342
Compliance with HBP (0% vs. over 0%)	1.602	(1.182, 2.172)	0.0024

*SBP* systolic blood pressure, *DBP* diastolic blood pressure, *FBS* fasting blood glucose, *BMI* body mass index, *eGFR* estimated glomerular filtration rate, *LDL-C* low-density lipoprotein cholesterol, *HDL-C* high-density lipoprotein cholesterol

^a^Target organ damage was defined as a composite of 1) stroke, transient ischemic attack, or vascular dementia; 2) left ventricular hypertrophy, angina, myocardiac infarction, or heart failure; 3) albuminuria (albumin level > 30 mg/g) or chronic kidney disease (eGFR < 60 mL/min/1.73 m^2^); 4) peripheral vascular disease (ankle-brachial index < 0.9), pulse wave velocity > 10 m/sec, intimal thickness of the carotid artery > 1.0 mm or large artery disease; and 5) stage 3 or 4 hypertensive retinopathy

^b^Dyslipidemia was defined as total cholesterol level ≥ 230 mg/dL, LDL-C level ≥ 150 mg/dL, HDL-C level < 40 mg/dL, or triglyceride ≥200 mg/dL

### Current status of HBP in Korea

The number of participants with sphygmomanometers in their home was significantly higher in the intervention group than in the control group (302 [24.01%] vs. 174 [14.22%], *p* < 0.001).

Among the 476 participants who had sphygmomanometers in their home, the number of participants who never measured BP at home was 166 (34.87%). Only 128 (26.89%) participants measured BP at least once a week for > 4 weeks. Among the patients (*n* = 128) who regularly measured BP, HBP was measured at a median of four times a week (minimum 1 to maximum 30).

Among the 2006 participants who have no sphygmomanometers in their home, 1563 (77.92%) participants responded that they do not consider purchasing home sphygmomanometers. In response to the question of “Why you did not buy a sphygmomanometer for your home?”, approximately 40.83% of patients did not think the HBP measurement was needed, and a similar percentage (37.04%) of participants were skeptical about their measurement capability or willingness to perform HBP measurements. Approximately 14.41% participants responded that they had difficulty in selecting a good model of sphygmomanometers, and only 7.63% of the participants did not purchase the device because of the price burden.

### Safety profile of fimasartan

Among the safety analysis group, 1.6% (*n* = 43:32 [1.96%] and 21 [1.24%] patients; 49 and 27 cases in the intervention and control group, respectively) experienced ADRs. The incidences of the most common ADRs, such as dizziness and headache, were 0.72 and 0.24%, respectively. The incidence of other ADRs, such as dyspepsia, flushing, asthenia, and orthostatic hypotension, was very rare, with a incidence of 0.09–0.12%. Among the ADRs, 86.84% (66/76 cases) were mild and 13.16% (10/76 cases) were moderate, and no SADRs occurred.

## Discussion

This study is the first to investigate the current status of HBP measurement and to comprehensively examine the effect of HBP measurement on BP control. We first established that subgroup that measured HBP at least once subgroup had positive effect on BP control compared to subgroup that has not measured HBP at all. However, the efficacy of introducing HBP measurements could not be proved through this study. Additionally, this study presented the various obstacles to HBP measurement among Korean patients. Further, in this study, fimasartan showed results for BP reduction efficacy and safety in hypertensive Korean patients similar to the findings of previous studies [24, 25].

According to prior literature, HBP is a more accurate prognostic indicator than conventional CBP, because of the greater number of measurements and the minimization of the white-coat effect [26]. Thus, HBP measurement could be incorporated into patient care and could be recommended in near future. Several meta-analysis studies have shown that, compared with usual care, the use of HBP measurement is associated with significant reduction in systolic and diastolic BP, as well as reduction in antihypertensive medication and therapeutic inertia, defined as unchanged medication despite elevated CBP [5, 27–29]. Although most studies have focused on the White population, this study has a value, as it validated the effect of HBP measurement on BP control in an Asian population, especially Koreans.

HBP measurement is known to be useful for promoting medication adherence, compliance, and lifestyle changes, helps make patients become more aware of their chronic condition, determines the efficacy of antihypertensive therapy, and supports appropriate adjustments [30]. However, in a previous meta-analysis, proactive additional support (counselling, education, behavioral management, medication management with decision, adherence contracts, and so on) to improve efficacy of HBP was proposed [31]. In our study, during the intervention process of this study, liberal HBP measurement could not improve BP control.

Our study has several limitations. First, although the study was conducted in patients with stage 2 hypertension or patients requiring ≥2 combination therapies, the CBP target was attained at 12 weeks by 64.15% and 68.73% of the patients in the intervention and control groups, respectively. Given that this is already a high CBP control attainment rate, HBP measurements may not have shown an additive effect. Second, HBP may not be effective for improving BP control rate due to the participants’ low compliance. Follow-up on HBP compliance could not be provided. Among the 1258 participants, 455 (36.2%), 572 (45.5%), and 659 (52.4%) did not measure HBP during the 4-, 8-, and 12-week follow-up, respectively. Compared to compliance in previous studies on HBP compliance and BP control (91.0% measured their BP at least > 12 times per week), the compliance in this study is much lower [32]. Third, we did not plan to survey positive effects of HBP other than BP control, such as improving medication adherence or awareness in patients or medication titration by physicians. Although HBP could not improve the BP control rate, measuring HBP may have been helpful for patients and physicians to manage hypertension in the long term. Fourth, because this study performed cluster randomization and the recruitment of patients was not stratified by their confounding variables for group assignment, there were statistically significant differences in various characteristics between the groups. Although demographic factors and potential confounders were statistically adjusted, un-collectable confounders may have influenced the results.

In the current HBP status in Korea, only 19.17% (*n* = 476) of the participants were found to have a home sphygmomanometer; of these, only one-third of these patients measured their BP at least once a week, whereas the other one-third did not measure at all. Besides, among the intervention group, the subgroup that measured HBP at least once compliance subgroup had better BP control than subgroup that has not measured HBP at all (66.36% vs. 56.95%, *p* = 0.003), indicating that actual compliance with HBP measurements, rather than only introducing HBP, may affect BP control. Therefore, for proper chronic disease management and control, repeatedly encouraging and educating the patients is essential to emphasize its' importance. To improve HBP measurement and treatment compliance, tele-transmission of BP using a home sphygmomanometer could be utilized, and further studies need to be conducted [33].

## Conclusions

The HBP measurement did not improve BP control, but better compliance with the HBP measurement improved the BP control rate. Moreover, fimasartan has favorable safety and effectiveness profiles in stage 2 hypertensive Korean patients or hypertensive patients who requiring 2 and more antihypertensive agents, including fimasartan.

## Supplementary information

**Additional file 1.** Inclusion and Exclusion Criteria of the Included Patients. Detailed inclusion and exclusion criteria are descripted.

**Additional file 2.** Home Blood Pressure (BP) Measurement Timing and Preparations. Detailed timing, preparations and method to measure home blood pressure are descripted.

**Additional file 3.** Data Collection Schedule During the Study Period. Description of data: Detailed data collection schedule and contents during the study period are descripted.

## Data Availability

The datasets are not publicly available but are available from the corresponding author upon reasonable request.

## Abbreviations

BP: Blood pressure
CBP: Clinic blood pressure
HBP: Home blood pressure
24-h ABP: 24-h ambulatory blood pressure
ADR: Adverse drug reactions
SADR: Serious adverse drug reaction
IRB: Institutional review board
