# Supplementary material for: Effect of home blood pressure monitoring for blood pressure control in hypertensive patients taking multiple antihypertensive medications including fimasartan (the FORTE study)
Source: Clin Hypertens. 2020 Dec 15;26:24. doi: 10.1186/s40885-020-00154-y (PMC7737356; doi:10.1186/s40885-020-00154-y)
Supplement: Supplementary file 1 — Additional file 1. Inclusion and Exclusion Criteria of the Included Patients. Detailed inclusion and exclusion criteria are descripted. [file 40885_2020_154_MOESM1_ESM.docx]

**Additional file 1: Inclusion and Exclusion Criteria of the Included Patients**

**(1) Inclusion criteria**

1. Those who signed the informed consent form after the purpose and methods of the study were explained to them.

2. Adult men and women aged ≥19 years

3. Those requiring the administration of at least ≥2 antihypertensive agents, including fimasartan* (blood pressure [BP] is measured twice at clinic and averaged).

*Those who satisfied the following criteria regardless of whether they were receiving fimasartan

- Those without antihypertensive agents administered within 4 weeks prior to enrollment (drug naïve): Patients with stage 2 hypertension (systolic BP [SBP] ≥160 mmHg or diastolic BP [DBP] ≥100 mmHg) or with stage 1 hypertension and higher (SBP ≥140 mmHg or DBP ≥90 mmHg) accompanied by target organ damage or cardiovascular diseases
- Those with hypertension uncontrolled by existing antihypertensive agents: Patients whose BP not reaching the target BP (SBP <140 mmHg and DBP <90 mmHg) despite being treated with existing antihypertensive agents for >4 weeks. However, for special cases below, the target BP must follow the criteria suggested:
- People aged ≥80 years: SBP <150 mmHg and DBP <90 mmHg
- Diabetic patients: SBP <140 mmHg and DBP <85 mmHg
- Patients with chronic kidney disease, as indicated by proteinuria (albumin level ≥30 mg/d or ≥30 mg/g [albuminuria], protein level ≥150 mg/d or ≥150 mg/g [proteinuria]): SBP <130 mmHg and DBP <80 mmHg

**(2) Exclusion criteria**

1. Patients on hemodialysis

2. Patients with diabetic nephropathy and those taking an angiotension-converting enzyme inhibitor

3. Patients with moderate-to-severe liver diseases (aspartate aminotransferase and alanine aminotransferase levels are 3-fold higher than the upper limit of normal or with biliary obstruction

4. Patients with galactose intolerance, Lapp lactase deficiency, or glucose-galactose malabsorption

5. Patients with hypersensitivity to ingredients contained in fimasartan

6. Pregnant or lactating women

7. Those currently participating in other clinical studies or planning to participate in other clinical studies while being in this study

8. Other cases judged by an investigator to be adversely affected by the study or unable to follow an investigator’s instructions

9. In the case of the control site, patients who planned to regularly measure HBP during the study period
