# Supplementary material for: Effect of home blood pressure monitoring for blood pressure control in hypertensive patients taking multiple antihypertensive medications including fimasartan (the FORTE study)
Source: Clin Hypertens. 2020 Dec 15;26:24. doi: 10.1186/s40885-020-00154-y (PMC7737356; doi:10.1186/s40885-020-00154-y)
Supplement: Supplementary file 2 — Additional file 2. Home Blood Pressure (BP) Measurement Timing and Preparations. Detailed timing, preparations and method to measure home blood pressure are descripted. [file 40885_2020_154_MOESM2_ESM.docx]

**Additional file 2. Home Blood Pressure (BP) Measurement Timing and Preparations**

(1) Morning: Prepare a BP diary and a pen and urinate within an hour after waking up and then measure BP twice at 2-minute intervals before having breakfast and before taking antihypertensive agents. BP was measured after taking a rest at least for 5 minutes in a sitting position and recorded along with the pulse rates

(2) Evening: Prepare a BP diary and a pen, and then measure BP twice at 2-minute intervals. BP was measured after taking a rest at least for 5 minutes in a sitting position and recorded along with the pulse rates.
