# Supplementary material for: Effect of home blood pressure monitoring for blood pressure control in hypertensive patients taking multiple antihypertensive medications including fimasartan (the FORTE study)
Source: Clin Hypertens. 2020 Dec 15;26:24. doi: 10.1186/s40885-020-00154-y (PMC7737356; doi:10.1186/s40885-020-00154-y)
Supplement: Supplementary file 3 — Additional file 3. Data Collection Schedule During the Study Period. Description of data: Detailed data collection schedule and contents during the study period are descripted. [file 40885_2020_154_MOESM3_ESM.docx]

**Additional file 3. Data Collection Schedule During the Study Period.**

| **Data collection schedule** ^1^ | **V1**  **(screening and enrollment)** | **V2**  **(4^th^ week after V1)** | **V3**  **(8^th^ week after V1)** | **V4**  **(12^th^ week after V1)** |
| --- | --- | --- | --- | --- |
| Signed informed consent forms^2^ | ● |  |  |  |
| Demographic information | ● |  |  |  |
| Medical history and cardiovascular risk factors^3^ | ● |  |  |  |
| Antihypertensive agents^4^ | ● | ● | ● | ● |
| Other concomitant agents/combination treatment^5^ | ● | ● | ● | ● |
| Inclusion/exclusion criteria checked | ● |  |  |  |
| Survey^6^ | ● |  |  |  |
| Height and weight ^7^ | ● | ● | ● | ● |
| CBP readings/CBP^8^ | ● | ● | ● | ● |
| Collection of HBP readings^9^ |  | ● | ● | ● |
| Laboratory findings^10^ | ○ | ○ | ○ | ○ |
| Antihypertensive agent-associated ADRs/SAEs |  | ● | ● | ● |

●Collected (collected during routine clinical practice after the baseline without requesting each participant to visit the center)

○Collected if any

^1^It is recommended to set visit schedules after V1 to 4^th^ week±1 week, 8^th^ week±1 week, and 12^th^ week±1 week from V1. Given that this is an observational study, the suggested schedules and tolerance (recommended dates±1 week) were used as reference; and if the clinical visit schedules do not coincide with the study visit schedules, an investigator scheduled a visit based on a participant’s clinical needs.

^2^Informed consent forms were collected within 14 days prior to the baseline (Day 0). All study activities were performed after the participants signed the consent forms, and the patients eligible to participate in the study were followed up.

^3^Medical history at 6 months from V1 was collected, and it was also checked if the participants have a target organ damage or cardiovascular risk factors.

^4^Data of antihypertensive agents were collected by dividing them into the preceding ones administered prior to the baseline (Day 0) and the ones administered during the study. Preceding antihypertensive agents are defined as agents administered from 4 weeks prior to the baseline (Day 28) to the day before the baseline (Day -1) (agents withdrawn during the period included). Information on drug names, fixed-dose antihypertensive combination/single agent, daily doses, and start/end dates of dosing were collected, and those administered for other purposes at a dose not influencing the control of BP were recorded as other concomitant drugs (in this case, data of agents administered from the baseline only were collected).

^5^Data of concomitant drugs administered from the baseline (Day 0) to the date of when the observation was completed (drug names, purposes, and start/end dates of dosing) and data of combination treatment (treatment name, purposes, and start/end dates of treatment) were collected. Combination treatment is defined as a procedure or operation performed as a medical intervention other than drug dosing to treat disease or relieve symptoms (i.e., diagnostic test results were not collected).

^6^The following information was checked on all participants on Visit 1.

- Presence of home sphygmomanometer: If participants currently have a sphygmomanometer that can measure BP at home was checked. If not, it was investigated whether the participant considered of buying one, and the reason of not purchasing the device was also investigated.
- Regular measurement of HBP: We checked whether the participants have ever measured BP at least once a week for 4 consecutive weeks within the last 3 months.

^7^After Visit 1, only weight was measured on Visits 2-4.

^8^When the participants visited the center, CBP was measured twice at 2-minute intervals after a 5-minute rest. The arm with higher mean systolic blood pressure by measuring BP in both arms twice for each on the baseline point was used as the reference arm (if systolic pressures are the same in both arms, diastolic blood pressure is used); and in subsequent visits, the BP and pulse rate measured twice on the reference arm were collected.

^9^Applicable to participants at test sites (measuring both CBP and HBP). Participants were instructed to measure BP twice a day (twice an hour after waking up and twice before sleeping at 2-minute intervals; total of four times) for 7 days from the scheduled visit and recorded the BP readings along with the pulse rates in the BP diary provided. After Visit 1, participants were reminded to measure HBP and record BP through SMS or calls (only if SMS is unavailable) 8-10 days prior to the study visits.

^10^Separate laboratory tests were not performed for this study, but the following data were collected if available: hemoglobin, blood urea nitrogen, creatinine, glucose, glycated hemoglobin, total bilirubin, aspartate aminotransferase, alanine aminotransferase, alkaline phosphatase, high sensitivity C-reactive protein, total cholesterol, high-density lipoprotein cholesterol, low-density lipoprotein cholesterol, triglyceride, and fasting blood glucose.
HBP, home blood pressure; CBP, clinic blood pressure; BP, blood pressure
